# Draft Genome Sequence of the Rodent Opportunistic Pathogen *Pasteurella pneumotropica* ATCC 35149^T^

**DOI:** 10.1128/genomeA.00771-14

**Published:** 2014-08-07

**Authors:** Hiraku Sasaki, Hiroki Ishikawa, Ryoki Asano, Hidehiro Ueshiba, Tetsuya Matsumoto, Ron Boot, Eiichi Kawamoto

**Affiliations:** a Department of Health Science, School of Health and Sports Science, Juntendo University, Chiba, Japan; b Department of Microbiology, Tokyo Medical University, Tokyo, Japan; c Department of Biotechnology, Faculty of Bioresource Sciences, Akita Prefectural University, Akita, Japan; d Institute of Laboratory Animals, Tokyo Women’s Medical University, Tokyo, Japan; e National Institute of Public Health and the Environment Bilthoven, the Netherlands; f Animal Research Center, Tokyo Medical University, Tokyo, Japan

## Abstract

*Pasteurella pneumotropica* is an opportunistic pathogen in rodents that is commonly isolated from upper respiratory tracts in laboratory rodents. Here, we report the draft genome sequence of the *P. pneumotropica* type strain ATCC 35149, which was first isolated and characterized as biotype Jawetz.

## GENOME ANNOUNCEMENT

*Pasteurella pneumotropica* is a Gram-negative rod-shaped bacterium that is an infectious agent of rodent pasteurellosis (1). *P. pneumotropica* infection causes various clinical symptoms in immunodeficient and immunosuppressed animals; however, the effects on health are not always observed in immunocompetent animals (2–4). Although *P. pneumotropica* belongs to the rodent cluster in *Pasteurellaceae*, this bacterium has not been formally classified taxonomically under the genus *Pasteurella* (5). Therefore, the whole-genome data of the type strain might not only contribute to our understanding of the pathogenic potential of this bacterium but also to its taxonomic position within the *Pasteurellaceae* family. In recent studies, *P. pneumotropica* outer membrane component proteins were used as vaccine immunogens against pathogens (6, 7). Thus, genome sequencing will allow us to exploit and improve these vaccine substances. In this study, we report the draft genome sequence of *P. pneumotropica* type strain ATCC 35149, which was generated using long-read next-generation sequencing.

The *P. pneumotropica* ATCC 35149 (= NCTC 8141) genome was sequenced using PacBio RS II, and the sequencing data were assembled *de novo* by using single-molecule real-time (SMRT) analysis (8). Insert libraries (3 to 10 kb) were sequenced from four SMRT cells, and approximately 200× genome coverage was extracted and used for the sequence assembly. The assembly generated 9 contigs, comprising a 2.43-Mb genome with 39.87% G+C content (average contig length, 270 kb; *N*_50_ length, 815 kb). Genome annotation was performed using the Microbial Genome Annotation Pipeline (http://www.migap.org/) (9). The coding sequences and ribosome binding sites were predicted using MetaGeneAnnotator, and tRNAs and rRNAs were predicted using tRNAscan-SE and RNAmmer, respectively (10, 11). In total, 2,243 coding regions and 66 tRNA and 6 rRNA operons were identified by performing genome annotation. With respect to putative virulence-associated genes, a 4th novel repeat-in-toxin (RTX)-like toxin-coding gene was identified in addition to the three known RTX toxin-coding genes (12, 13). Of these, multiple copies of *pnxIIA* were present in the ATCC 35149 genome. Hemagglutination and hemolysis are reported virulence-related phenotypes of *P. pneumotropica* (14, 15). In ATCC 35149, a number of genes encoding hemagglutination- and hemolysis-associated proteins were predicted to be secreted via the type V secretion pathway. We identified the gene encoding filamentous hemagglutination. The sequence of this gene is partially similar to that of *ibpA* in *Histophilus somni* as well as *pfhB1* and *pfhB2* identified in *Pasteurella multocida*, gene products thought to have essential roles in bacteria cell attachment and invasion (16). Moreover, strain ATCC 35149 has genes encoding type VI secretion system proteins. The type VI secretion system effector Hcp family proteins are possibly involved in the pathogenicity of *P. pneumotropica* (17).

### Nucleotide sequence accession numbers.

The draft genome sequence of *P. pneumotropica* ATCC 35149 has been deposited in DDBJ/EMBL/GenBank under the accession numbers BBIX01000001 to BBIX01000009. The version mentioned in this study is the noncorrected first version.
